# The New York Institute of Stomatology

**Published:** 1905-02

**Authors:** 

**Affiliations:** The New York Institute of Stomatology


### Reports of Society Meetings.

### THE NEW YORK INSTITUTE OF STOMATOLOGY.

A MEETING of the Institute was held at the "Chelsea," No. 222 West Twenty-third Street, New York, November, 1904, the President, Dr. A. H. Brockway, in the chair.

The minutes of the last meeting were read and approved.

The regular business of the evening was opened by Dr. J. F. P. Hodson, who presented several useful appliances and methods.

*Dr. Hodson.*—I shall present this evening a number of little helps that are familiar to me in my practice, but some of which may be new to you.

*First.*—A combination of flexible steel holding point, with ball and socket jointed mirror, to hold in the left hand while starting gold fillings in deeply placed approximal and posterior cavities, until the fillings shall have become self-anchored.

*Second.*—A little thin, saucer-like attachment to be slipped over the back and edge of the mouth-mirror for catching pieces of amalgam as it is being carried into a cavity, or bits of gold while cutting out a gold stopping, instead of letting them drop into the mouth or on the dam. It can be slipped on or off from the mirror in an instant. Every operator should accustom himself to operating in the mirror, held in the left hand to reflect the operation; it transforms difficult of access, posterior cavities into easy ones, and even when not so needed, is still of great value in the left hand for throwing a ball of light upon the work.

*Third.*—An old engine hand-piece that has been locked, making an excellent socket for a hand bur.

*Fourth.*—For very many years I have had manufactured for me the round cedar sticks for cleansing teeth. I still use them for that purpose, and I have found, when they have worn short, that a convenient way of lengthening them is to put them into an ordinary penholder with the pen part removed.

*Fifth.*—A pair of amalgam carriers, that I use more than all my other amalgam carriers put together, one for forward and the other for backward work.

*Sixth.*—In connection with amalgam, some know, and some do not seem to, how to trim the margins of amalgam stoppings under the gum, judging from the ragged masses which we often see hardened under the gum. The best instrument for that purpose is a thin, round, flexible, old-fashioned canal-plugger, which, carried along that edge, will clean the margin perfectly, and, followed with a thin, flat burnisher, will leave as perfect edges under the gum as above it.

*Seventh.*—A mouth-mirror with the glass broken out makes an excellent scoop for catching up bits of amalgam or gold from the rubber dam.

*Eighth.*—A rubber apron, that I always adjust the moment the patient gets into the chair. A so-called improvement has been made upon mine by some one, consisting of a pocket to catch the saliva and hold it. I think that is merely filthy. With this apron the patient's clothes are kept neat and clean from drops of water, saliva, etc. The patient is made comfortable with the thought that her clothes will not be spotted. The white towel is of course placed over it. I get this material by the yard at a very small price, and all the work of making them consists in sewing on the neck tape.

*Ninth.*—To go back to amalgam stoppings, for finishing them off and wiping margins I use a piece of wet spunk instead of cotton, as it gives a much finer grain and is much smoother.

*Tenth.*—The paper points for drying root-canals that I presented to you a good many years ago are an illustration of a principle that I apply as such very largely in my practice,—the principle of capillary attraction. It is astonishing what can be done by means of it. In drying out root-canals a piece of cotton on a broach will push material ahead of it, possibly carrying the septic material beyond the apex of the root, while these paper points made from bibulous paper will attract the moisture and absorb it instead of driving it ahead. Syringing a root-canal will never cleanse it, but three or four floodings alternated with the paper point absorbings will certainly do so. The points are made from the French bibulous paper. They are also sold by Johnson & Johnson.

*Eleventh.*—The principle of capillary attraction suggests conditions allied thereto. For instance, I never find it necessary to make retaining points in building amalgam on to an old amalgam stopping for the purpose of repairing it. It is sufficient to first make the surface of the old filling, in that part where the repair is to be made, perfectly bright and clean, and then tinning that surface with a portion of very thinly mixed amalgam. The new amalgam takes perfect hold of this, being a cold soldered joint.

*Twelfth.*—I also use this principle in filling thin, winding root-canals, and, in fact, all others as well. I fill my root-canals with chloropercha and gutta-percha. By wetting a fine smooth broach with the chloropercha and carrying it to the end of these small canals, and in this position touching it with a drop of chloropercha, capillary attraction will surely carry it to the end of the canal. This, however, is in my hands only the preparation for the root-filling. The chloropercha is merely to cement the gutta-percha point that I stick to the end of a nerve-canal instrument and gently carry to the end of the canal. The root is thus filled surely to the end, no air-bubbles, and the minimum of the chloropercha.

*Thirteenth.*—Many years ago Dr. Merriam showed us a method of making gutta-percha stoppings stick in wet cavities by taking a piece of gutta-percha on the proper instrument, warming it, and carrying it to the cavity immediately after having dipped it in oil of cajuput. As the surface of such gutta-percha stoppings will not wear well, it is best to use this method only for very temporary stoppings or for the first part of a stopping where it is intended to dry out the remainder of the cavity and fill with a stronger gutta-percha. I have sometimes filled the collar-like cavities around the necks of teeth with a modification of this method, drying out the cavity as well as possible, then touching with the oil of cajuput and applying the stopping. It is astonishing how well the gutta-percha will stick with this method.

*Fourteenth.*—Another bit of advising born of my own experience as a patient. When I was a boy student a dentist who at that time stood in the very front rank for ability and skill, while condensing a gold filling in my mouth, brought all his power upon the instrument held *off hand,* and the instrument slipped and went through my cheek. Since which time I have never made even the smallest move in the mouth which was not guarded by a fulcrum or rest, formed by the third finger end on the teeth for fingers grasp, and by the thumb for the fist grasp of the instrument, and I can truthfully say that in consequence I have never in my life let an instrument slip to hurt a patient.

*Fifteenth.*—Speaking of slipping instruments reminds me of a matter somewhat akin to it. It may happen at very rare intervals, even with the most skilful operator, that in the hurried changing of an instrument during an operation he may catch it in the rubber dam and make a rent. The need for a stopper is most instant and urgent. I suggested such a remedy many years ago. It consists of a section sawed from a cork, filed oval on both sides, and a deep groove running all round the edge, thus making it like two thin oval buttons placed back to back. A half-dozen sizes kept at hand will fit all possible rents, and the proper-sized one quickly stretched therein will effectually dam out all moisture,—will hold its place perfectly and all without being in the way, even though it be in the immediate vicinity of the filling.

*Dr. G. W. Weld.*—I wish first to mention one little point in connection with Jenkins's little tray for fusing inlays. The trays that come with the Jenkins outfit are composed of some composition other than platinum, and repeated heating quickly burns them out. I have remedied this by riveting to the bottom of the little pan a platinum disk. I find this will stand the heat splendidly. It will be necessary to put five rivets in; this makes practically a platinum pan.

What I wish to show specially is home-made impression cups for porcelain inlays. The subject is not entirely new, and these cups have been placed upon the market, but I have found them of little practical value to the dentist. We all know that the foundation of a perfect inlay is a correct impression or a correct matrix, or both. If the matrix can be taken directly from the cavity it will save time, and is often done. I have done it myself, and have had beautiful fits. But there are thousands of cases where this cannot be done. But with a correct model of the cavity set in a piece of plaster it is very easy to tease the matrix away from it, whether it be of gold or platinum. Therefore, when I can I use an impression. It follows that to take an impression of a cavity you must have a proper cup to do it with. Take, for instance, two approximal cavities between the central incisor teeth, where the front plate of enamel is intact but extremely thin. Porcelain inlays are indicated here for the reason that there is great danger in fracturing the enamel walls in putting in a gold filling. A right and left impression cup is here needed. They are made of platinoid, but can be made from German silver. A little piece of the platinoid is cut out the proper size and shape, and to this is soldered another piece of the same material to serve as a handle. In this way a perfect impression cup can be made, using the smallest quantity of impression material possible; a surplus being as bad as an insufficiency. The impression is then set with the convex surface upward in a little plaster, and after that has hardened the surface is painted with talcum powder. Stiff Harvard or Ames cement is then pressed down on the impression and allowed to remain for some time. When hard, the model thus made is set in a piece of plaster. Then the gold can be easily teased away without injuring its shape.

The most simple cavities are the cavities on the labial surfaces of the front teeth. Here it is easy to take the matrix directly from the cavity. Indeed, there are cases of compound cavities where I have succeeded better with this direct method. Sometimes it is better not to make the little holes in the cup, designed to hold the material better, thus allowing the cup to be removed first, and taking out the impression afterwards.

*Dr. W. D. Tracy.*—Inasmuch as this society seemed last year to take such an active interest in orthodontia, I thought it would not be amiss to show some little instruments I have devised to assist in the adjustment of the Angle bands. The set consists of three instruments, which I shall pass around together with a band fitted upon a model. The conditions on the model are somewhat exaggerated for illustration. The working face of the instruments are finely serrated, so that they will not slip when used with heavy pressure to carry this band in contact with the tooth.

Dr. Hodson spoke of trimming the cervical margin of amalgam fillings with a canal-plugger.

In my own experience something more rigid than the instrument shown by Dr. Hodson is necessary, because the amalgam, being packed quite dry, hardens quickly. To meet the requirements of trimming all kinds of fillings on the approximal surface I have had made this pair of right and left trimmers, which I submit to your criticism.

*Dr. S. H. McNaughton.*—I wish to present some two-angled excavators—hatchets and hoes—which have been very useful to me. In the preparation of cavities in the approximal surfaces of teeth in which plastic fillings are to be inserted, and where it is desirable to save as much enamel as possible as a protection to the filling, by means of these instruments all the angles may be reached and all the decay removed without the necessity of much loss of enamel. The width of blade is three-, four-, or five-tenths of a millimetre, the length of blade three times the width; the first angle (the angle nearest the cutting edge) is twenty-five centigrades (ninety degrees), the second angle is twelve and five-tenths centigrades (forty-five degrees)—and should be the length of blade from the first angle; the direction at this angle should be to the left, or to the right, or in same direction as at first angle. These constitute the three shapes and sizes which are indispensable to me.

In filling large occlusal cavities where one or both approximal surfaces are involved, where the walls are frail and badly broken away, especially lower molars, I make use of a combination, filling in the following manner: I use a matrix made from a section of a ping-pong ball. Instead of first placing zinc phosphate over the floor and partly on the buccal and lingual walls, as is ordinarily done, I first pack amalgam against the gingival wall, the matrix to the occlusal edge, and the margins of the buccal and lingual walls. When this has been done I have a cavity with four walls and a floor, which may be filled two-thirds or entirely with zinc phosphate. If but partially filled with the zinc phosphate, it may be completed with amalgam, in which case it has the appearance of an all-amalgam filling. The advantages of a filling of this kind are: The amalgam is keyed in by the cement; it is held while setting in perfectly close contact with the walls, so much so that in removing one of these fillings it seems to adhere to the walls as tightly as does a copper amalgam plug. There will be no washing out at the gingival margin, as is likely to be with zinc phosphate; it holds its shape better than gutta-percha. There are other classes of cavities in which this method or variations of it may be used.

As an investment for soldering I use a mixture of equal parts of modelling clay, coarse powdered pumice, and ground asbestos, which I keep wet in a fruit-jar, by which means it is always ready. It is especially good for small pieces, and it holds very tightly. It does not set. As a support for it a piece of coarse iron wire netting is used. Wax will be burnt off in the heating.

*Dr. F. L. Fossume.*—Right lower central incisor missing, and patient wearing a small rubber plate with an artificial substitute. This rubber plate impinged on the gums and lingual surfaces of the necks of the teeth which were denuded. The teeth were slightly loose but otherwise in perfect condition.

I inserted a dummy in the following manner: The central and lateral incisors on either side of the space were ground lingually in the centre at the cutting edge, forming a hollow into which pure gold 40-gauge was burnished. Rivet holes were bored near the wall approximating the space. Platino-iridium wire about the thickness of the pin in a rubber tooth was forced through the gold into the holes and bent so as to meet the backing of the dummy to be inserted. The gold lining and pin in position, they were tacked together with hard wax and soldered; then they were placed back on the teeth and an impression taken in plaster. The dummy was now ground, backed, and waxed into place. The whole piece was invested and the backing soldered to the rivet wire and gold, with 22-carat solder. Solder was allowed to flow on the gold only along and over the platino-iridium wire. The cavities prepared in the cutting edges were now undercut at sides distally to the approximating space, where the rivet holes were bored. After polishing the backing, the piece was cemented into place and the thin gold hammered into the undercut and built up with gold-foil the same as in any other cavity. The whole thing was then polished and burnished; the only gold visible was a small half-moonshaped filling on the cutting edge on the central and lateral on either side of the dummy.

*Dr. F. Milton Smith.*—Previous to entering upon the discussion of these various subjects, I have the privilege of presenting to the Institute some radiographs sent me by Dr. C. Edmund Kells, of New Orleans.

While Dr. Kells did not send these for exhibition, I have taken the liberty of bringing them here, that you may get an inkling of Dr. Kells's work in this line.

*Dr. J. Bond Littig.*—I can verify Dr. McNaughton's statement with regard to asbestos and clay as an investment. I have used for many years a little ordinary moulding sand with asbestos. I put it upon a piece of wire gauze and put the piece to be soldered right into it, and without waiting for it to thoroughly harden heat it up and let the moisture dry out. In ten minutes I can have my piece soldered. I have, as I say, been doing this for many years, and you can find it published in "Catching's Compendium," 1890.

*Dr. Chas. O. Kimball.*—I have lately encountered a case where the X-ray was of great assistance. A little girl about nine years old presented with a left lateral in a very markedly crooked condition. When we came to get a radiograph of the condition, it was found that the canine on that side, which was yet up in the tissues, was misplaced and was bearing in an abnormal way upon the root of this lateral, causing the peculiar irregularity. It is easy to imagine what my difficulty would have been had I tried to regulate that tooth without knowing the condition causing the irregularity.

*Dr. F. Milton Smith.*—I wish to express my extreme gratitude to Dr. Hodson for the comfort and help I have derived from the combination mirror he has shown us to-night. I have used it, more or less, for twenty years.

*Dr. F. C. Brush.*—Regarding investments for soldering, I have found that a combination of two-thirds plaster and one-third ashes is excellent. It makes a very hard investment that will not change in shape or crack under the heat of the blow-pipe. The ashes should be in the form of the fine powder obtained from ordinary coal ashes.

Upon motion of Dr. F. Milton Smith, a vote of thanks was extended to the gentlemen who so kindly assisted in the helpful entertainment of the evening.

Adjourned.

FRED. L. BOGUE, M.D., D.D.S.,
*Editor The New York Institute of Stomatology.*

